# The accuracy of spectral CT with quantitative parameters in differentiating septic from aseptic periprosthetic complications

**DOI:** 10.1186/s13244-026-02265-w

**Published:** 2026-04-07

**Authors:** Zhangyan Xu, Suying Zhou, Jie Zhao, Tongxin Zhu, Bo Sheng, Sadaqat Ali, Wei Zeng, Yongliang Pu, Haitao Yang

**Affiliations:** 1 Department of Radiology, The First Affiliated Hospital of Chongqing Medical University, No.1 Youyi Road, Yuzhong District, 400016 Chongqing, China; 2 Department of Radiology, People’s Hospital of Guang’an City, No. 1, Section 4, Binhe Road, Chengnan, 638000 Guang’an City, Sichuan China; 3 Imaging and Nuclear Medicine, Chongqing Medical University, No.1 Yixueyuan Road, Yuzhong District, 400016 Chongqing, China

**Keywords:** Spectral CT, Iodine concentration, Periprosthetic joint infection, Quantitative parameters

## Abstract

**Objectives:**

To explore the accuracy of spectral CT, using quantitative parameters, in differentiating septic from aseptic periprosthetic complications.

**Materials and methods:**

This study consecutively included patients with clinically suspected periprosthetic complications who underwent preoperative spectral CT and planned revision surgery between September 2020 and June 2024. Spectral quantitative parameters, including iodine concentration (IC), effective atomic number (Zeff), and normalized iodine concentration (NIC) on arterial and venous phases (AP, VP) for periprosthetic bone and soft tissue lesions were measured. Qualitative assessment of periprosthetic joint infection (PJI) was conducted by reviewing spectral CT images. The diagnostic performance of quantitative parameters for PJI was evaluated and compared to qualitative assessment.

**Results:**

A total of 59 patients with 62 prostheses (mean age ± SD, 67.20 years ± 12.12; 26 men; septic group: aseptic group 26: 36 prostheses) were included. The IC-AP values were significantly higher in the septic group compared to the aseptic group across integrated and separate analyses of bone and soft tissue lesions (1.25 ± 0.59 vs. 0.50 ± 0.36; 1.58 ± 0.74 vs. 0.53 ± 0.37; and 1.10 ± 0.43 vs. 0.43 ± 0.32, respectively; all *p* < 0.001). IC-AP showed the highest diagnostic accuracy, with values of 84.3% (integrated), 90.7% (bone), and 89.6% (soft tissue). Other parameters also showed significant differences (all *p* ≤ 0.012) and good diagnostic performance. The diagnostic accuracy of qualitative assessment by the senior and junior radiologists, based on CT imaging features of PJI, was 82.3% and 75.8%, respectively.

**Conclusions:**

Spectral CT with quantitative parameters has the potential to improve the differentiation between septic and aseptic periprosthetic complications.

**Critical relevance statement:**

Spectral CT may offer quantitative imaging biomarkers with the potential to improve visualization and identification of periprosthetic joint infections, particularly in complex cases and among less-experienced radiologists, thereby facilitating the clinical decision-making process.

**Key Points:**

- Accurate diagnosis of periprosthetic joint infection is crucial for treatment decisions.
- Quantitative parameters of spectral CT showed significant increases and high diagnostic accuracy in identifying septic periprosthetic complications.
- Spectral CT with quantitative parameters may serve as an imaging biomarker to enhance the clinical decision-making process.

**Graphical Abstract:**

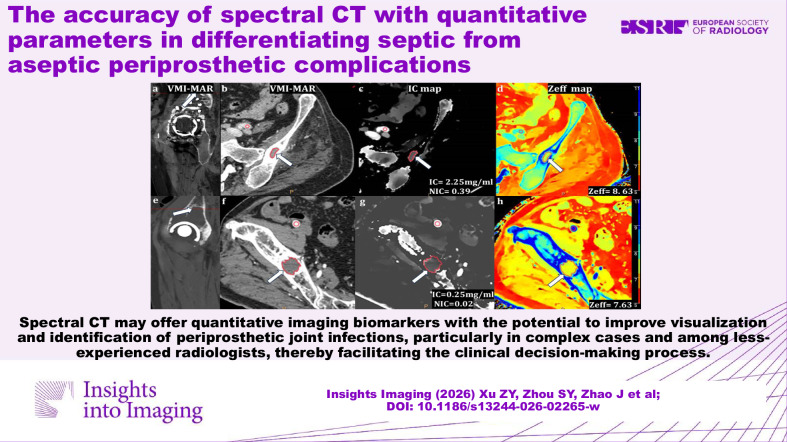

## Introduction

Total knee and hip arthroplasty (TKA and THA), serving as the most effective therapeutic option for advanced osteoarthritis, has been performed widely and proliferated in recent decades [1, 2]. The outcomes are commonly excellent, providing significant pain relief and functional restoration. Despite the efficacy of arthroplasty, periprosthetic complications (e.g., aseptic loosening, infection, fractures, liner wear) occur on occasion; nationwide data spanning 2005–2014 indicate a general incidence of 1.96% in the post-THA patient population [3]. Periprosthetic joint infection (PJI) is one of the most destructive and common reason of joint arthroplasty failure, often leading to second or even multiple revisions [4, 5]. Differentiating between septic and aseptic periprosthetic complications is essential due to the distinct treatment strategies and prognoses associated with each condition. PJI conventionally requires lengthy hospitalization, prolonged antimicrobial treatment, and even repeated surgical interventions. These patients typically experience higher medical costs and poorer outcomes, including increased risks of reinfection, amputation, systemic complications, and even mortality [6, 7].

No single laboratory or imaging test can reliably diagnose PJI due to limited sensitivity and/or specificity [8, 9]. The most frequently used PJI definitions, the 2018 International Consensus Meeting (ICM) and the 2021 European Bone and Joint Infection Society (EBJIS), combine multiple tests but generally exclude imaging techniques except for white blood cell scintigraphy [10, 11]. MRI metal artifact reduction sequences can mitigate metal artifacts and improve image quality, and several MRI features help distinguish septic from aseptic complications [12–14]. However, these assessments were based on subjective imaging features.

Conventional CT is limited by the presence of metal-induced artifacts. Spectral CT allows for virtual monoenergetic imaging (VMI) reconstructions at a high kilovoltage level. Several studies have demonstrated that VMI and orthopedic metal artifact reduction (O-MAR) post-processing algorithms can improve image quality by reducing metal artifacts in the periprosthetic bone and soft tissues [15, 16]. Dual-energy computed tomography (DECT) has been shown to achieve higher diagnostic performance than single-energy CT and radiography in evaluating periprosthetic loosening after TKA and THA, and demonstrates diagnostic performance comparable to that of MRI for detecting musculoskeletal infections [17–20]. Additionally, spectral CT offers multiple quantitative parameters, such as the iodine concentration (IC) and effective atomic number (Zeff), that enhance lesion conspicuity and elevate diagnostic accuracy [21]. Studies have demonstrated that DECT quantitative parameters aided in discerning infection and assessing inflammatory activity [22–24].

Differentiating septic from aseptic complications is clinically critical, as it directly informs optimal management and improves patient outcomes. Conventional imaging modalities offer limited utility due to prosthesis-induced artifacts and overlapping imaging features. Spectral CT provides quantitative parameters beyond metal artifacts reduction, potentially enhancing diagnostic confidence in PJI and supporting clinical decision-making. Septic and aseptic periprosthetic complications arise from distinct pathophysiology. Given that DECT quantitative parameters have exhibited valuable potential for identifying infections and material decomposition in other diseases [23, 25], we hypothesized that these spectral parameters could aid in differentiating septic from aseptic periprosthetic complications. The purpose of this study is to investigate the accuracy and reliability of spectral CT with quantitative parameters in distinguishing PJI.

## Methods

### Participants

**Fig. 1:**
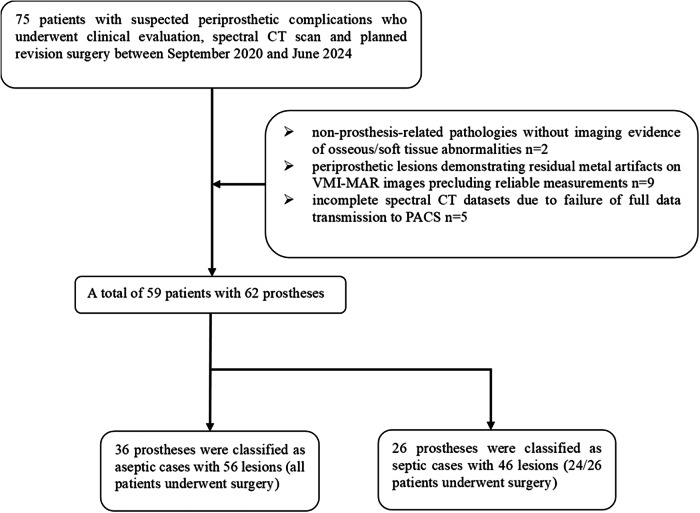
Study flowchart for the inclusion and exclusion criteria of patients

This ambispective cohort study was approved by the Institutional Review Board in June 2021 (Approval No. 2021-272). Data collected from September 2020 to June 2021 were reviewed retrospectively with waived informed consent, while from June 2021 to June 2024, patients were included prospectively with written informed consent. Patients with suspected periprosthetic complications were consecutively included, presenting at the orthopedic department, which consists of two medical groups. All patients underwent comprehensive clinical evaluations, preoperative spectral CT scans, and planning for revision surgery. Exclusion criteria comprised: (1) non-prosthesis-related pathologies without imaging evidence of osseous/soft tissue abnormalities, (2) periprosthetic lesions demonstrating residual metal artifacts on VMI-MAR images precluding reliable measurements, and (3) incomplete spectral CT datasets due to failure of full data transmission to PACS. Thirty participants were reported in a previous study on the optimal visualization of both periprosthetic vessels and metal artifact reduction by spectral CT [16]. The study flow diagram is depicted in Fig. 1.

### Clinical assessment and diagnosis of septic and aseptic complications

All participants underwent a comprehensive assessment, including a medical history, physical examination, serological and synovial analysis, radiographic evaluation, organism culturing, and histological examination. Experienced orthopedic surgeons performed revision surgeries via the prior incision. Upon surgical entry, they assessed prosthesis stability, wear, and the presence and severity of purulent material or infected tissue. Intraoperative frozen-section analysis was performed to evaluate neutrophilic infiltration. A one-stage revision was performed in aseptic cases after removing hyperplastic tissues. For septic cases, infected tissues were debrided, and a decision was made regarding either one-stage or two-stage revision based on the severity of the infection. Histopathological and microbiological evaluations were conducted on the collected samples. Postoperative antibiotic protocols varied based on infection status.

The 2018 ICM criteria were utilized as the reference standard for diagnosing PJI [11]. Meeting each of the following major criteria indicated definite PJI: (1) the presence of a sinus tract with communication to the joint or visualization of the prosthesis, (2) two positive cultures of the same organism. Additionally, various minor criteria with weighted values were employed to assess failed joint arthroplasty. A cumulative score (combining preoperative biomarkers and intraoperative findings) of ≥ 6 was indicative of infection, while scores of 4–5 were inconclusive, and scores ≤ 3 suggested the absence of infection. Not meeting diagnostic criteria for PJI, patients were classified as aseptic periprosthetic complications, including aseptic loosening, liner wear, dislocation, and periprosthetic fracture during revision surgery.

### Spectral CT scan protocol

CT scans of the affected joints (unilateral or bilateral knee joints, or bilateral hip joints) were performed using a spectral-detector CT (IQon, Philips Healthcare). Detailed scan parameters included a pitch of 0.391, a collimation of 64 × 0.625 mm, a rotation time of 0.5 s, a matrix size of 512 × 512, a tube voltage of 120 kVp, a scan slice thickness of 5 mm, and a reconstruction thickness of 0.8 mm. Automated tube current modulation (3D dose modulation, DoseRight Index) was applied with an index of 24 (reference mAs, 162 mAs). All patients underwent non-contrast and contrast-enhanced spectral CT scans. Intravenous contrast material (370 mg I/m, Iopromide, Ultravist, Bayer Healthcare) was administered at 1.5 mL/kg (dose) and 3.0 mL/s (rate) per standard protocol. For special patients (underweight, obese, renal dysfunction, or poor vascular access), dose and rate were adjusted to 1–2 mL/kg and 2.5–4.0 mL/s, respectively. Arterial phase (AP) and venous phase (VP) images were acquired with fixed delays of 40 seconds and 70 seconds after contrast agent administration, respectively. Conventional images were computed using a hybrid iterative reconstruction algorithm (iDose 4, level 3; Philips Healthcare). Spectral-based images (SBI) were calculated using a dedicated spectral reconstruction algorithm (Spectral, level 3; Philips Healthcare). Both conventional images and SBI were post-processed with or without the O-MAR algorithm (O-MAR, Philips Healthcare). The SBI-MAR was utilized to reconstruct the IC map and Zeff map using a dedicated image viewer (IntelliSpace Portal, Philips Healthcare).

### Spectral CT quantitative image analysis

**Fig. 2:**
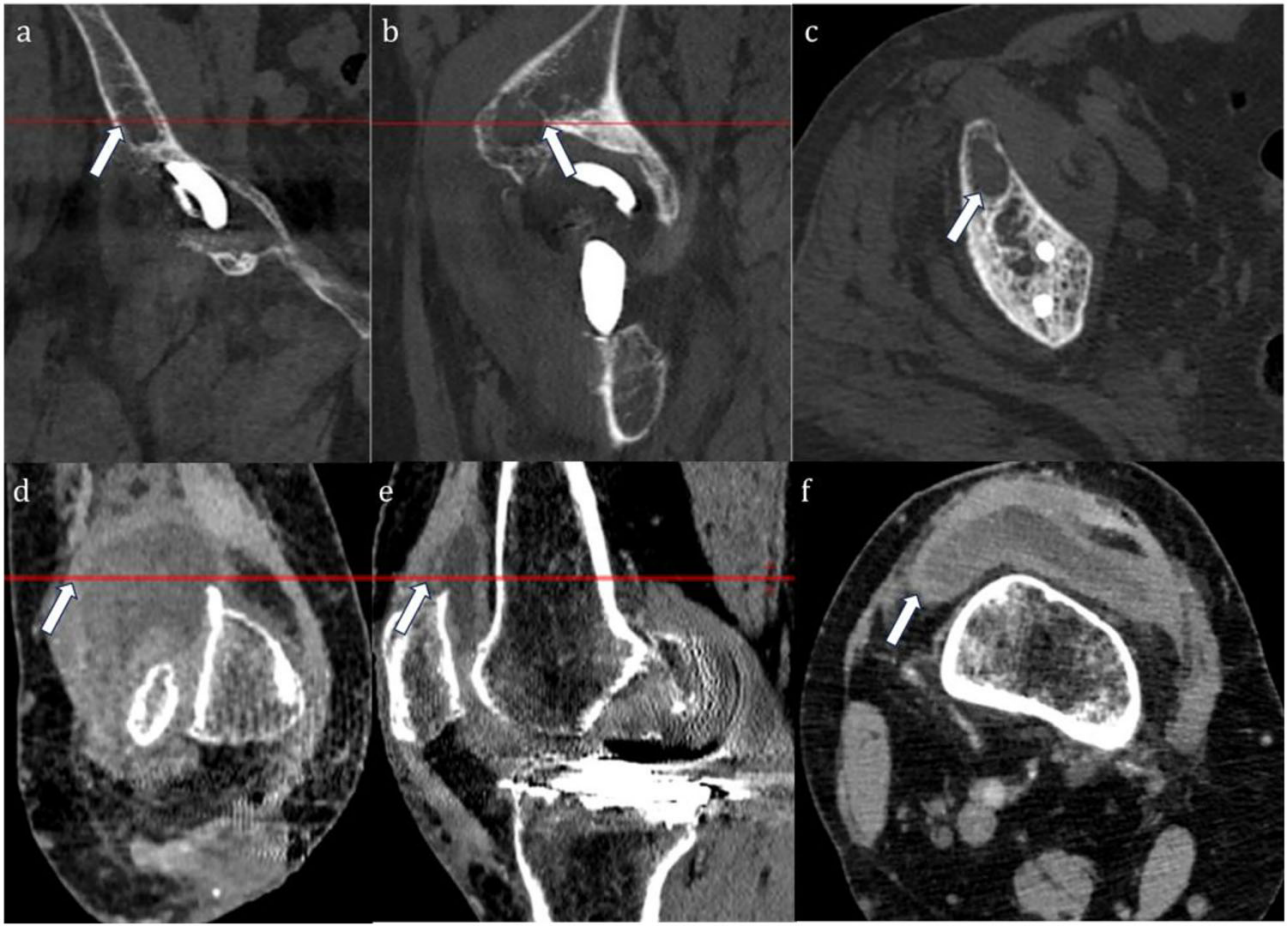
Illustration of bone (**a**–**c**) and soft (**d**–**f**) tissue lesions localization. The virtual monochromatic combined with orthopedic metal artifact reduction (VMI-MAR) images were reconstructed using multiplanar reformation. Coronal (**a**, **d**) and sagittal (**b**, **e**) images show periprosthetic bone and soft tissue lesions (arrows). Axial image (**c**, **f**) shows bone and soft tissue lesions (arrows) without metal artifact obscuration

**Fig. 3:**
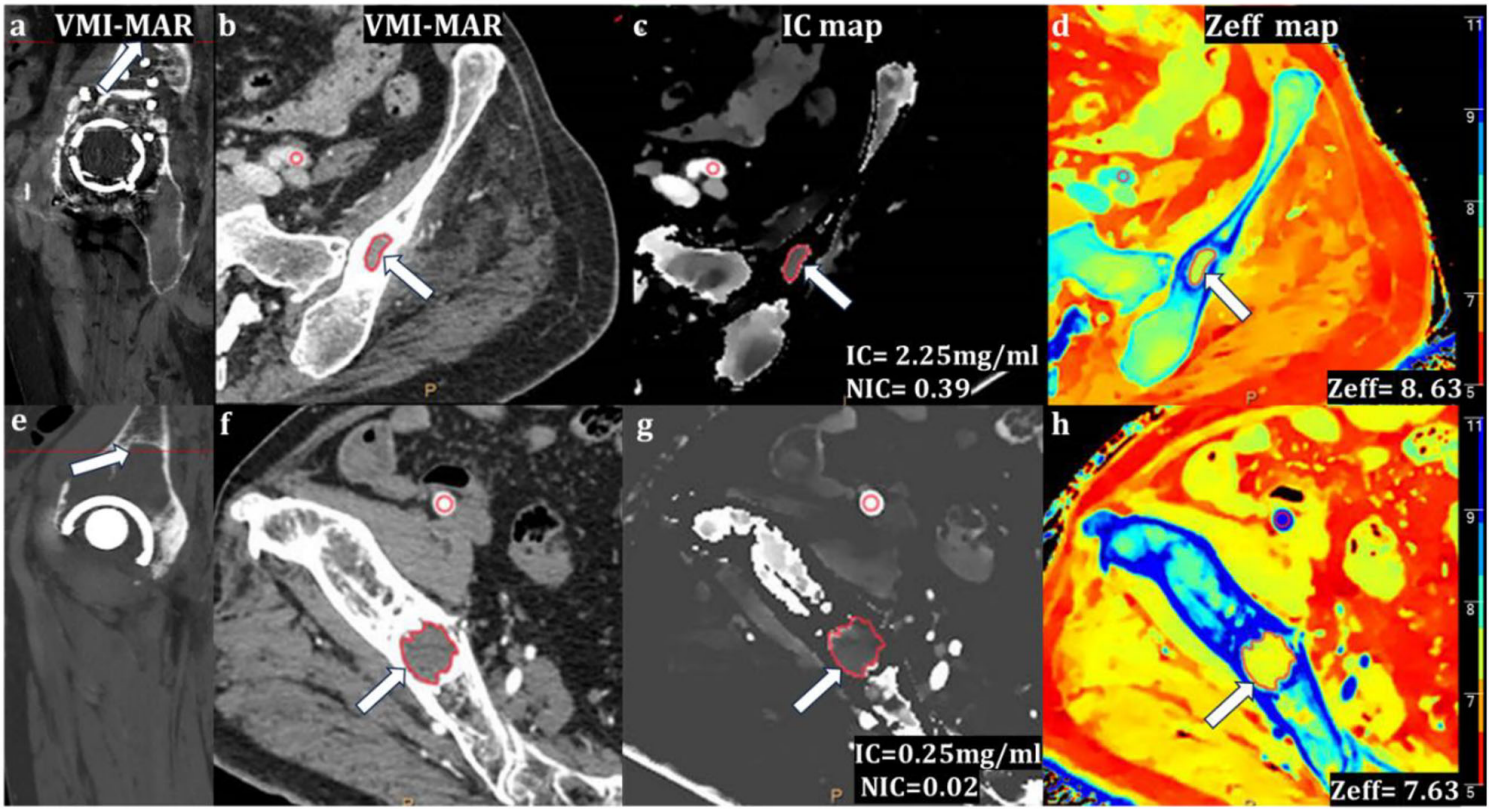
Images show examples of spectral CT for evaluation of bone lesions (arrows) in septic and aseptic cases during the arterial phase. Sagittal (**a**, **e**) and axial (**b**, **f**) virtual monochromatic combined with orthopedic metal artifact reduction (VMI-MAR) images; axial iodine concentration map (**c**, **g**); axial effective atomic number map (**d**, **h**). Freehand ROIs were drawn on periprosthetic bone/soft tissue lesions, and an additional circle ROI was placed on an adjacent artery for normalization. A 56-year-old female was diagnosed with periprosthetic joint infection (**a**–**d**) with periprosthetic osteolytic lesion (iodine concentration during the arterial phase (IC-AP) = 2.25 mg/mL, effective atomic number during the arterial phase (Zeff-AP) = 8.63, the normalized iodine concentration during the arterial phase (NIC-AP) = 0.39). A 73-year-old male was classified as an aseptic periprosthetic complication (**e**–**h**) with a periprosthetic osteolytic lesion (IC-AP = 0.25 mg/mL, Zeff-AP = 7.63, NIC-AP = 0.02)

**Fig. 4:**
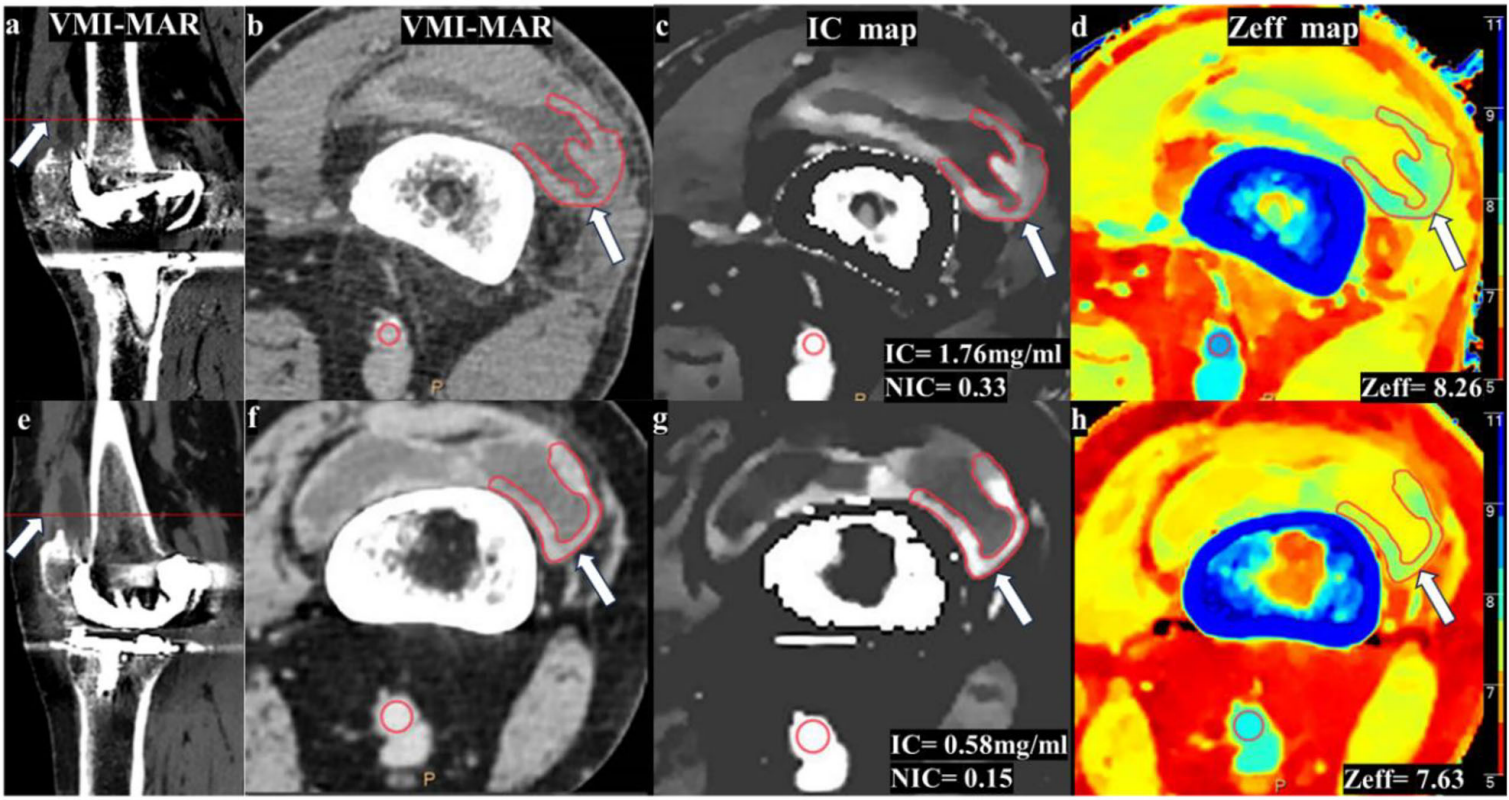
Images show examples of spectral CT for evaluation of soft tissue lesions (arrows) in septic and aseptic cases during the venous phase. Sagittal (**a**, **e**) and axial (**b**, **f**) virtual monochromatic combined with orthopedic metal artifact reduction (VMI-MAR) images; axial iodine concentration map (**c**, **g**); axial effective atomic number map (**d**, **h**). Freehand ROIs were drawn on periprosthetic bone/soft tissue lesions, and an additional circle ROI was placed on an adjacent artery for normalization. A 71-year-old male was diagnosed with periprosthetic joint infection (**a**–**d**) with proliferative synovial tissue (iodine concentration during venous phase (IC-VP) = 1.76 mg/mL, effective atomic number during venous phase (Zeff-VP) = 8.26, the normalized iodine concentration during venous phase (NIC-VP) = 0.33). A 69-year-old female was classified as an aseptic periprosthetic complication (**e**–**h**) with proliferative synovial tissue (IC-VP = 0.58 mg/mL, Zeff-VP = 7.63, NIC-VP = 0.15)

Energy levels were adjusted, and images were reconstructed via free multiplanar reformation to optimize the visualization of periprosthetic lesions, avoiding metal artifacts interference. All lesions (bone: osteolysis; soft tissue: proliferative synovial tissue, abscess cavity, sinus tract, inflammatory pseudotumor) were evaluated on the VMI-MAR images (Fig. 2), with the lesions’ definition referring to previous literature [26]. Freehand region of interest (ROI) was manually delineated along the edges of osteolytic lesions and the enhanced soft tissue lesions without obscuration of metal artifacts on axial VMI-MAR images (120 KeV). The same ROI was automatically copied to the IC map and Zeff map for calculation of the average IC and Zeff (Figs. 3 and 4). A circular ROI was placed on the artery (iliac artery, femoral artery, or popliteal artery) at the same level, and the normalized iodine concentration (NIC) was defined as IC_lesion_/IC_artery_. If the lesion was unobscured by artifacts across multiple levels, the consecutive measurements were averaged. The same protocol was applied to each independent lesion if multiple lesions existed. Consistency in the position, shape, and size of the ROIs was maintained on AP and VP images.

Blinded to clinical information, all quantitative measurements were performed by two junior radiologists (Xu and Zhao, with 2 and 3 years of experience in musculoskeletal radiology and spectral CT imaging interpretation, respectively) and reassessed after a 4-week interval (Xu). The final decision was made by a senior musculoskeletal radiologist (Yang, with 15 years of experience in musculoskeletal radiology) in cases of disagreement between two junior radiologists regarding whether metal artifacts obscured the bone/soft tissue lesions. Before quantitative measurements, the two juniors received training in lesion identification, artifact recognition, and standardized measurement procedures.

### Spectral CT qualitative image analysis

Two radiologists (Sheng and Zhu, a senior and a junior musculoskeletal radiologist with 14 and 3 years of clinical experience, respectively) independently reviewed the spectral CT images processed by VMI-MAR on the dedicated image viewer (IntelliSpace Portal, Philips Healthcare). Each radiologist subjectively evaluated the presence of infection based on the CT imaging features (sinus, soft tissue abscesses, fluid accumulation in periprosthetic muscle and fat tissue, periosteal reaction), previously reported to be indicative of infection [27, 28], and documented the diagnosis of each case (PJI, aseptic cases such as loosening, periprosthetic fracture, dislocation, osteolysis, inflammatory pseudotumor, and liner wear).

### Statistics analysis

To compare the clinical findings and spectral CT quantitative parameters between septic and aseptic groups, the Chi-square test/Fisher’s exact test was used for categorical variables, and the two-tailed t-test/Mann-Whitney U-test was used for continuous variables. The Spearman test was employed to evaluate the correlation between PJI and quantitative parameters. Receiver operating characteristic (ROC) analysis was conducted, and the area under the ROC curve (AUC) was calculated to evaluate the diagnostic performance of quantitative parameters in identifying PJI. The maximum Youden index was used to determine the optimal cutoff value. Accuracy, sensitivity, specificity, positive predictive value (PPV), and negative predictive value (NPV) were computed. Subgroup analysis was performed based on lesion type (bone or soft tissue lesions). The Chi-square test was used to calculate the sensitivity, specificity, PPV, NPV, and accuracy of qualitative assessments. Inter- and intra-observer agreement was assessed with Intra-class correlation coefficients (ICCs) for spectral quantitative parameters and Kappa analysis for qualitative assessments. The ICC was classified as poor (< 0.40), fair (0.40–0.59), good (> 0.60–0.74), and excellent (0.75–1.00), and the *κ*-values was defined as poor (< 0.20), fair (0.21–0.40), moderate (0.41–0.60), substantial (0.61–0.80), excellent (0.81–1.00). Statistical analyses were conducted with IBM SPSS Statistics software (IBM Corp. Released 2020. IBM SPSS Statistics for Windows, Version 27.0. Armonk, NY: IBM Corp). A *p*-value of less than 0.05 was considered statistically significant, and adjustments for multiple comparisons were implemented using the Bonferroni correction.

## Results

### Participant characteristics

A total of 75 patients with suspected periprosthetic complications were initially enrolled, of whom 16 were excluded based on the predefined exclusion criteria, resulting in a final study cohort of 59 participants (mean age ± SD, 67.20 years ± 12.12; 26 men) with 62 prostheses. Based on the 2018 ICM criteria, 36 prostheses were classified as aseptic cases, and 26 prostheses were classified as septic cases (Fig. 1). The demographic characteristics of all included patients are presented in Table 1. No significant differences were found in age, sex, and body mass index. The affected hip or knee joints in the two groups showed significantly different distributions (*p* = 0.012).

**Table 1:** Demographic and clinical data of the participants

Clinical data	Aseptic group	Septic group	*p-*value
Age	67.12 ± 13.04 (34–88)	67.31 ± 11.11 (32–84)	0.879
Sex (M)	16 (44.4%)	10 (38.5%)	0.638
BMI	24.06 ± 4.32 (17.19-36.67)	25.59 ± 3.22 (19.15–33.33)	0.123
Affected joints (THA)	31 (86.1%)	15 (57.7%)	0.012
Elevated CRP	8 (22.2%)	20 (76.9%)	< 0.001
Elevated ESR	20 (55.6%)	21 (80.8%)	0.038
Successful aspiration	9 (25.0%)	13 (50.0%)	0.042
Elevated synovial PMN % (> 80%)	1 (11.1%)	10 (76.9%)	0.008
Intraoperative frank pus	0 (0.0%)	16 (66.7%)	< 0.001
Positive frozen section (> 5 WBCs per high power field)	0 (0.0%)	15 (62.5%)	< 0.001
Successful obtaining of samples for culture	23 (63.9%)	26 (100.0%)	< 0.001
Positive microbiologic culture	0 (0.0%)	12 (46.2%)	< 0.001
Sinus tract	0 (0.0%)	9 (34.6%)	< 0.001

Data are means ± SDs (minimum- maximum) or number (%)

*M* male, *BMI* body mass index, *THA* total hip arthroplasty, *CRP*   C-reactive protein, *ESR* erythrocyte sedimentation rate, *PMN* polymorphonuclear leukocytes, *WBC* white blood cell

The septic cases consisted of 15 THAs and 11 TKAs. The diagnostic evidence was as follows: sinus tract (9 cases), two positive cultures of the same organism (8 cases), and a cumulative score ≥ 6 (14 cases). Five cases had both sinus tracts and two positive cultures simultaneously. Eight cases had two positive cultures with the identical microbiological findings, and four cases had only one positive culture result. The details of microbiological findings are shown in supplementary table S1. Among the 26 septic prostheses, 16 underwent a two-stage revision, 7 had a one-stage revision, 1 underwent debridement only, and two patients refused a surgical intervention and were treated only with antibiotics. The aseptic cases comprised 31 THAs and 5 TKAs; the final diagnosis was periprosthetic loosening (30 cases, with three cases having concomitant inflammatory pseudotumor) and liner wear/dislocation (6 cases). Among the 36 aseptic cases, 23 cases had negative microbiological cultures. The remaining 13 cases did not have specimens collected for culture; however, they displayed no signs of infection based on intraoperative exploration and histopathological diagnosis. All aseptic cases underwent one-stage revision surgery.

### Comparisons of quantitative analysis between the septic and the aseptic group

In the integrated analysis including all bone and soft tissue lesions, the mean value of IC-AP, NIC-AP, Zeff-AP, IC-VP, NIC-VP, and Zeff-VP in the septic group was 1.25 ± 0.59, 0.15 ± 0.11, 8.00 ± 0.31, 1.57 ± 0.58, 0.43 ± 0.22, 8.16 ± 0.28; and they were 0.50 ± 0.36, 0.06 ± 0.05, 7.55 ± 0.34, 0.71 ± 0.45, 0.23 ± 0.20, 7.71 ± 0.29 in the aseptic group. All the quantitative parameters of the septic group were significantly higher than those of the aseptic group (all *p* < 0.001). The same results were obtained regarding the separate analyses of both bone lesions (all *p* ≤ 0.001) and soft tissue lesions (all *p* ≤ 0.012). The results were illustrated in Table 2.

**Table 2:** Difference of quantitative parameters between the septic group and the aseptic group

All lesions	Quantitative parameters	Septic group (*n* = 46)	Aseptic group (*n* = 56)	*T*/*Z*	p*-value
	IC-AP	1.25±0.59	0.50 ± 0.36	−6.93^b^	< 0.001
	Zeff-AP	8.00 ± 0.31	7.55 ± 0.34	−6.84^a^	< 0.001
	NIC-AP	0.15 ± 0.11	0.06 ± 0.05	−5.96^b^	< 0.001
	IC-VP	1.57 ± 0.58	0.71 ± 0.45	−6.85^b^	< 0.001
	Zeff-VP	8.16 ± 0.28	7.71 ± 0.29	−8.06^a^	< 0.001
	NIC-VP	0.43 ± 0.22	0.23 ± 0.20	−5.41^b^	< 0.001
**Subgroup I: Bone lesions**	**Quantitative parameters**	**Septic group (*n*** = **15)**	**Aseptic group (*n*** = **39)**	**T/Z**	p*-value
	IC-AP	1.58 ± 0.74	0.53 ± 0.37	−4.85^b^	< 0.001
	Zeff-AP	8.15 ± 0.41	7.56 ± 0.39	−4.89^a^	< 0.001
	NIC-AP	0.20±0.15	0.06 ± 0.05	−4.43^b^	< 0.001
	IC-VP	1.78 ± 0.80	0.66 ± 0.42	−4.59^b^	< 0.001
	Zeff-VP	8.26 ± 0.38	7.68 ± 0.30	−5.83^a^	< 0.001
	NIC-VP	0.48 ± 0.28	0.22 ± 0.21	−3.72^b^	0.001
**Subgroup II: Soft lesions**	**Quantitative parameters**	**Septic Group (*n* = 31)**	**Aseptic group (*n* = 17)**	**T/Z**	p*-value
	IC-AP	1.10 ± 0.43	0.43 ± 0.32	−4.35^b^	< 0.001
	Zeff-AP	7.92 ± 0.23	7.52 ± 0.20	−4.34^b^	< 0.001
	NIC-AP	0.12 ± 0.06	0.05 ± 0.04	−3.49^b^	0.002
	IC-VP	1.47 ± 0.41	0.81 ± 0.50	−3.66^b^	< 0.001
	Zeff-VP	8.11 ± 0.21	7.75 ± 0.28	−3.57^b^	0.001
	NIC-VP	0.41 ± 0.19	0.25 ± 0.18	−3.15^b^	0.012

Data are mean ± standard deviation

a = the two-tailed T Test, b = Mann–Whitney *U*-Test, *n *= the number of lesions

*IC* iodine concentration, *Zeff* effective atomic number, *NIC* the normalized iodine concentration, *AP* arterial phase, *VP* venous phase

p*-value was adjusted with Bonferroni correction

### Correlation analysis and diagnostic performance

For the integrated bone and soft tissue lesions, moderate correlations between spectral CT quantitative parameters and the presence of PJI were observed (*r* = 0.539–0.689, all *p* < 0.001), with IC-AP showing the strongest association. In the separate analyses of bone or soft tissue lesions, the correlations were slightly weaker than those of the integrated analysis (bone: *r *= 0.511–0.666, *p* < 0.001; soft tissue: *r* = 0.470–0.648, *p* < 0.001, respectively). The results are shown in Table 3.

**Table 3:** Results of the Spearman correlation coefficient of spectral quantitative parameters and the presence of periprosthetic joint infection

Quantitative parameters	Group		
	All lesions	Bone lesions	Soft lesions
IC-AP	0.689^*^	0.666^*^	0.648^*^
Zeff-AP	0.611^*^	0.564^*^	0.648^*^
NIC-AP	0.593^*^	0.609^*^	0.505^*^
IC-VP	0.681^*^	0.630^*^	0.543^*^
Zeff-VP	0.647^*^	0.593^*^	0.533^*^
NIC-VP	0.539^*^	0.511^*^	0.470^*^

*IC* iodine concentration, *Zeff* effective atomic number, *NIC* the normalized iodine concentration, *AP* arterial phase, *VP* venous phase

^*^Data indicate a value of *p* < 0.001

**Fig. 5:**
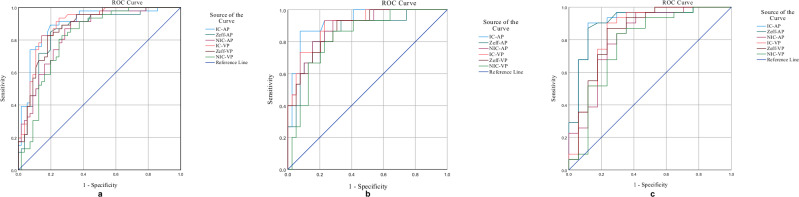
ROC curves of spectral CT quantitative parameters during arterial and venous phases for distinguishing septic from aseptic cases. **a** Integrated bone and soft tissue lesions. **b** Separate bone tissue lesions. **c** Separate soft tissue lesions. IC, iodine concentration; Zeff, effective atomic number; NIC, normalized iodine concentration; AP, arterial phase; VP, venous phase

Figure 5 and Table 4 present the ROC analyses of all spectral quantitative parameters for differentiating septic from aseptic lesions. Across integrated, bone-only, and soft-tissue-only analyses, AUCs ranged from 0.78 to 0.93, with accuracies between 75.9% and 90.7%. In the integrated analysis including both bone and soft-tissue lesions, IC-AP achieved the highest diagnostic performance (AUC = 0.90, 95% CI: 0.84–0.96; accuracy = 84.3%; sensitivity = 89.1%; specificity = 80.4%; PPV = 78.8%; NPV = 90.0%) at a threshold of 0.76 mg/mL. For bone lesions alone, IC-AP also showed the highest performance (AUC = 0.93, 95% CI: 0.86–1.00; accuracy = 90.7%; sensitivity = 86.7%; specificity = 92.3%; PPV = 81.3%; NPV = 94.7%) with a threshold of 0.89 mg/mL. For soft-tissue lesions alone, both IC-AP and Zeff-AP demonstrated the highest AUCs (both AUC = 0.91, 95% CI: 0.81–1.00), with IC-AP (threshold = 0.68 mg/mL; accuracy = 89.6%; sensitivity = 88.2%; specificity = 90.3%; PPV = 83.3%; NPV = 93.3%) and Zeff-AP (threshold = 7.72; accuracy  = 87.5%; sensitivity = 88.2%; specificity = 87.1%; PPV = 78.9%; NPV = 93.1%). A detailed confusion matrix for all quantitative parameters is provided in Table S2.

**Table 4:** The diagnostic performance of quantitative parameters between the septic group and the aseptic group

All lesions	Quantitative Parameters	AUC (95% CI)	Optimal threshold	Sensitivity (%)	Specificity (%)	PPV (%)	NPV (%)	Accuracy (%)
	IC-AP	0.90 (0.84–0.96)	0.76	89.1	80.4	78.8	90.0	84.3
	Zeff-AP	0.86 (0.78–0.93)	7.76	84.8	80.4	78.0	86.5	82.4
	NIC-AP	0.84 (0.77–0.92)	0.07	91.3	67.9	70.0	90.5	78.4
	IC-VP	0.89 (0.83–0.96)	0.85	93.5	75.0	75.4	93.3	83.3
	Zeff-VP	0.88 (0.81–0.94)	7.97	82.6	82.1	79.2	85.2	82.4
	NIC-VP	0.81 (0.73–0.90)	0.27	82.6	73.2	71.7	83.7	77.5
**Subgroup I: Bone lesions**	**Quantitative parameters**	**AUC (95% CI)**	**Optimal threshold**	**Sensitivity (%)**	**Specificity (%)**	**PPV (%)**	**NPV (%)**	**Accuracy (%)**
	IC-AP	0.93 (0.86–1.00)	0.89	86.7	92.3	81.3	94.7	90.7
	Zeff-AP	0.86 (0.75–0.98)	7.82	80.0	84.6	66.7	91.7	83.3
	NIC-AP	0.89 (0.80–0.98)	0.07	93.3	71.8	56.0	96.6	77.8
	IC-VP	0.91 (0.82–0.99)	0.89	93.3	76.9	60.9	96.8	81.5
	Zeff-VP	0.88 (0.79–0.98)	7.80	93.3	69.2	53.8	96.4	75.9
	NIC-VP	0.83 (0.71–0.95)	0.24	86.7	71.8	54.2	93.3	75.9
**Subgroup II: Soft lesions**	**Quantitative parameters**	**AUC (95% CI)**	**Optimal threshold**	**Sensitivity (%)**	**Specificity (%)**	**PPV (%)**	**NPV (%)**	**Accuracy (%)**
	IC-AP	0.91 (0.81–1.00)	0.68	88.2	90.3	83.3	93.3	89.6
	Zeff-AP	0.91 (0.81–1.00)	7.72	88.2	87.1	78.9	93.1	87.5
	NIC-AP	0.82 (0.68–0.96)	0.04	58.8	96.8	90.9	81.1	83.3
	IC-VP	0.85 (0.72–0.99)	0.87	76.5	90.3	81.3	87.5	85.4
	Zeff-VP	0.84 (0.70–0.98)	7.88	76.5	87.1	76.5	87.1	83.3
	NIC-VP	0.78 (0.62–0.93)	0.27	70.6	83.9	70.6	83.9	79.2

*IC* iodine concentration, *Zeff* effective atomic number, *NIC* the normalized iodine concentration, *AP* arterial phase, *VP* venous phase, *PPV* positive predictive value, *NPV* negative predictive value

In the qualitative assessment using VMI-MAR images, the senior radiologist achieved a sensitivity of 84.6%, a specificity of 80.6%, a PPV of 75.9%, a NPV of 87.9%, and an accuracy of 82.3% (51/62 cases). The junior radiologist obtained a sensitivity of 76.9%, a specificity of 75.0%, a PPV of 69.0%, a NPV of 81.8%, and an accuracy of 75.8% (47/62 cases). These subjective assessments were less accurate than the quantitative evaluation using IC-AP, which yielded accuracies of 84.3%, 90.7%, and 89.6% in integrated and separate analyses of bone/soft tissue lesions, respectively. The confusion matrix of qualitative assessments was provided in Table S3.

### Intra- and inter-observer reliability

Inter- and intra-observer reliability for quantitatively assessing bone and soft tissue lesions during AP and VP were excellent. During the AP, inter- and intra-observer reliability of the bone lesions assessment, with ICCs of 0.946 and 0.976, were higher than those of the soft tissue lesions, with ICCs of 0.895 and 0.895. During the VP, inter- and intra-observer reliability of the bone lesions assessment, with ICCs of 0.903 and 0.927, were higher than those of the soft tissue lesions, with ICCs of 0.860 and 0.880. The details were in Table S4. The inter-observer reliability for qualitative assessments between senior and junior radiologists was substantial (*κ *= 0.611).

## Discussion

Accurate diagnosis and proper treatment planning are key to improving prognosis, but still face challenges due to nonspecific and subtle findings among clinical manifestations, laboratory tests, and conventional images. This study investigated the discriminative value of spectral CT scan for suspected septic and aseptic periprosthetic complications. The findings showed that spectral CT with quantitative parameters can effectively distinguish septic from aseptic periprosthetic complications, and may provide additional imaging biomarkers for PJI identification, particularly in complex cases and among less-experienced radiologists.

Compared to aseptic periprosthetic complications, this study found that all spectral quantitative parameters were significantly increased in septic lesions and moderately correlated with the presence of PJI, especially IC-AP and IC-VP. Previous studies have verified that IC is associated with micro-vessel density, vascular maturation, and distribution, confirming this as a marker of vascularity [2, 25, 29]. The elevated IC in PJI may be related to enhanced vascularization, driven by active angiogenesis and a more abundant blood supply in septic cases. While aseptic loosening, a major cause of aseptic periprosthetic complications, is considered to result from released particle-induced foreign body granulomas, eventually leading to osteolysis and failed biological fixation [30]. The Zeff is derived from accurately analyzing substances with similar densities and close CT values [31]. In the present study, Zeff measured in septic cases was significantly higher than in aseptic cases, possibly indicating variations in biochemical compositions and blood supply.

This study showed that spectral CT quantitative parameters exhibited good to high diagnostic performance in differentiating septic from aseptic lesions (AUC: 0.78-0.93), with IC-AP yielding the highest accuracy in both integrated and separate bone/soft tissue lesions analyses. In subgroup analysis, the IC-AP of bone lesions showed slightly higher diagnostic accuracy (90.7%) compared to soft tissue lesions (89.6%). In PJI, microorganisms initially adhere to the prosthesis surface, proliferate, and form into biofilm that sustains infection [32]. Bone adjacent to the prosthesis inevitably exhibits earlier and more pronounced infection-related changes. Additionally, dense bone tends to localize infection to focal areas, while loose soft tissue facilitates the spread of infection to adjacent regions, potentially reducing the accuracy of quantitative parameters in capturing infection-related changes. Furthermore, the accuracy of IC-AP in integrated analysis (84%) was somewhat lower than in the subgroup analyses of separate bone (90.7%) and soft tissue (89.6%) lesions. The integrated analysis included all bone and soft tissue lesions, creating a larger, more heterogeneous sample of tissue characteristics and more variable quantitative parameters. This may reduce diagnostic performance compared to smaller, more homogeneous subgroups. The NIC was considered a more objective parameter, as it mitigates the influence of contrast agent amount, blood flow, and body weight. Although NIC showed slightly lower accuracy than IC in the present study, it still exhibited high AUC and accuracy in differentiating PJI from aseptic cases.

In the qualitative assessment of this study, the senior and junior radiologists achieved 82.3% and 75.8% accuracy, respectively, with substantial inter-observer agreement (*κ* = 0.611). This suggests that challenges persist in the subjective diagnosis of PJI using CT images, particularly among less-experienced radiologists. Previous studies have demonstrated that imaging modalities hold limited diagnostic value for PJI, primarily due to the overlap of imaging features and variation in radiologists’ clinical experience [33, 34]. This study suggested that spectral CT quantitative parameters may provide additional value, potentially serving as objective and reproducible imaging biomarkers to enhance diagnostic confidence of PJI.

In this cohort, elevated ESR, CRP, and PMN% were observed in the aseptic group, and the positive biomarkers in the septic group were limited to a range of 46.2% to 80.8% (Table 1), which is consistent with previous studies [35, 36]. Current clinical indicators, including serum and synovial fluid markers, culture, histopathology, and molecular testing, displayed variable sensitivity (42%–94%) and specificity (20%–100%) [9, 37, 38]. This study showed that spectral CT quantitative parameters achieved high diagnostic accuracy (75.9% to 90.7%) for differentiating septic from aseptic lesions. This indicates that spectral CT not only enhances the visualization of periprosthetic structures but also aids in identifying PJI via quantitative parameters. Thus, this technique can optimize clinical diagnosis protocol, for instance, reducing unnecessary joint aspirations and guiding precise pathological sampling.

There were limitations to this study. First, owing to the inclusion criteria and the relatively low incidence of PJI, the small sample size and imbalanced distribution between septic and aseptic groups may compromise the stability of the ROC analysis. Second, comparisons between clinical indicators and quantitative parameters were not conducted, nor was a multivariable model developed, due to the limited sample size. Third, the lesions were carefully selected to be sufficiently distant from metal, avoiding metal artifact interference and ensuring measurement accuracy. In the future, a larger multicenter database will be established to further validate the diagnostic efficiency of spectral CT quantitative parameters and develop combined multivariable models. Furthermore, photon-counting CT may offer unique advantages for PJI, owing to its superior metal artifact reduction, exceptional spatial resolution, and quantitative capability [39, 40].

In conclusion, this study demonstrates that spectral CT may provide quantitative imaging biomarkers to enhance diagnostic confidence in identifying PJI, particularly in complex cases and among less-experienced radiologists, thereby supporting and facilitating the clinical decision-making process.

## Supplementary information

ELECTRONIC SUPPLEMENTARY MATERIAL

## Data Availability

The datasets used and/or analyzed during the current study are available from the corresponding author on reasonable request.

## Abbreviations

AP: Arterial phase
AUC: Area under the curve
CRP: C-reactive protein
DECT: Dual energy computed tomography
ESR: Erythrocyte sedimentation rate
IC: Iodine concentration
ICCs: Intra-class correlation coefficients
ICM: International Consensus Meeting
NIC: Normalized iodine concentration
NPV: Negative predictive value
O-MAR: Orthopedic metal artifact reduction
PJI: Periprosthetic joint infection
PMN: Polymorphonuclear leukocytes
PPV: Positive predictive value
ROC: Receiver operating characteristics
ROI: Region of interest
SBI: Spectral-based images
THA: Total hip arthroplasty
TKA: Total knee arthroplasty
VMI: Virtual monochromatic imaging
VP: Venous phase
Zeff: Effective atomic number
