# Supplementary material for: The accuracy of spectral CT with quantitative parameters in differentiating septic from aseptic periprosthetic complications
Source: Insights Imaging. 2026 Apr 7;17:93. doi: 10.1186/s13244-026-02265-w (PMC13057172; doi:10.1186/s13244-026-02265-w)
Supplement: Supplementary file 1 — ELECTRONIC SUPPLEMENTARY MATERIAL [file 13244_2026_2265_MOESM1_ESM.pdf]

The Accuracy of Spectral CT with Quantitative Parameters in Differentiating Septic from Aseptic Periprosthetic Complications

ELECTRONIC SUPPLEMENTARY MATERIAL

Table S1: Microbiological Findings of Septic Group.

Culture result category	Pathogen	N
**Two positive cultures with identical results**	*Staphylococcus aureus*	3
	Staphylococcus epidermidis	1
	Enterococcus faecium	1
	Mycobacterium fortuitum	1
	Finegoldia magna	1
	Mixed flora (*Acinetobacter baumannii* + *Candida parapsilosis*)	1
**Single positive culture**	*Staphylococcus epidermidis*	2
	Pseudomonas aeruginos	1
	Mixed flora (*Staphylococcus aureus* + *Candida parapsilosis*)	1

Note. N= Number of cases

Table S2: Confusion matrix of spectral CT quantitative parameters for differentiating between the septic and the aseptic lesions

	Quantitative Parameters	TP	TN	FP	FN
**All Lesions (septic/aseptic= 46/56)**	IC-AP	41	45	11	5
	Zeff-AP	39	45	11	7
	NIC-AP	42	38	18	4
	IC-VP	43	42	14	3
	Zeff-VP	38	46	10	8
	NIC-VP	38	41	15	8
	**Quantitative Parameters**	**TP**	**TN**	**FP**	**FN**
**Subgroup I: Bone Lesions (septic/aseptic= 15/39)**	IC-AP	13	36	3	2
	Zeff-AP	12	33	6	3
	NIC-AP	14	28	11	1
	IC-VP	14	30	9	1
	Zeff-VP	14	27	12	1
	NIC-VP	13	28	11	2
	**Quantitative Parameters**	**TP**	**TN**	**FP**	**FN**
**Subgroup II: Soft Lesions (septic/aseptic= 31/17)**	IC-AP	15	28	3	2
	Zeff-AP	15	27	4	2
	NIC-AP	10	30	1	7
	IC-VP	13	28	3	4
	Zeff-VP	13	27	4	4
	NIC-VP	12	26	5	5

Note. - IC = iodine concentration, Zeff = effective atomic number, NIC = the normalized iodine concentration, AP = arterial phase, VP = venous phase, TP = true positive, TN = true negative, FP = false positive, FN = false negative

Table S3: Confusion matrix of qualitative assessments by two radiologists for differentiating septic from aseptic cases

	TP	TN	FP	FN
Radiologist 1	22	29	7	4
Radiologist 2	20	27	9	6

Note. - TP = true positive, TN = true negative, FP = false positive, FN = false negative

Table S4: The Results of Inter- and Intra-observer Reliability

	All-AP	Bone-AP	Soft-AP	All-VP	Bone-VP	Soft-VP
**Intra -**	0.950 (0.927-0.966)	0.976 (0.958-0.986)	0.895 (0.811-0.942)	0.918 (0.880-0.944)	0.927 (0.877-0.958)	0.880 (0.786-0.934)
***p*-Value**	＜0.001	＜0.001	＜0.001	＜0.001	＜0.001	＜0.001
**Inter-**	0.926 (0.861-0.961)	0.946 (0.865-0.979)	0.895 (0.742-0.959)	0.901 (0.816-0.948)	0.903 (0.766-0.961)	0.860 (0.664-0.945)
***p*-Value**	＜0.001	＜0.001	＜0.001	＜0.001	＜0.001	＜0.001

Note. - Data in parentheses are 95% CIs. AP = arterial phase, VP = venous phase
